# Sex-specific effect of P2Y_2_ purinergic receptor on glucose metabolism during acute inflammation

**DOI:** 10.3389/fendo.2023.1248139

**Published:** 2023-08-28

**Authors:** Randi J. Ulbricht, Christian A. Rivas, Hailee Marino, Erin Snyder, Dana James, Jamila Makhloufi, Nathan Johnson, Scott Zimmerman, Jianjie Wang

**Affiliations:** Department of Biomedical Sciences, Missouri State University, Springfield, MO, United States

**Keywords:** purinergic signaling, glucose homeostasis, P2Y receptor, inflammation, sex-dependence

## Abstract

The sex of an animal impacts glucose sensitivity, but little information is available regarding the mechanisms causing that difference, especially during acute inflammation. We examined sex-specific differences in the role of the P2Y_2_ receptor (P2Y_2_R) in glucose flux with and without LPS challenge. Male and female wild-type and P2Y_2_R knockout mice (P2Y_2_R^-/-^) were injected with LPS or saline and glucose tolerance tests (GTT) were performed. P2Y_2_R, insulin receptor, and GLUT4 transporter gene expression was also evaluated. Female mice had reduced fasting plasma glucose and females had reduced glucose excursion times compared to male mice during GTT. P2Y_2_R^-/-^ males had significantly decreased glucose flux throughout the GTT as compared to all female mice. Acute inflammation reduced fasting plasma glucose and the GTT area under the curve in both sexes. While both wild-type and P2Y_2_R^-/-^ male animals displayed reduced fasting glucose in LPS treatment, female mice did not have significant difference in glucose tolerance, suggesting that the effects of P2Y_2_R are specific to male mice, even under inflammatory conditions. Overall, we conclude that the role for the purinergic receptor, P2Y_2_R, in regulating glucose metabolism is minimal in females but plays a large role in male mice, particularly in the acute inflammatory state.

## 1 Introduction

The purinergic P2Y_2_ receptor (P2Y_2_R) is a G-protein coupled receptor that responds to extracellular nucleotides. Extracellular nucleotides, including ATP and UTP, activate P2Y_2_R, regulating renal function, fibrosis, neural development, and inflammatory processes, including leukocyte recruitment (1, 2). Notably, the P2Y_2_ receptor has been implicated in various inflammatory disease processes such as atherosclerosis, obesity, and diabetes (3). Under inflammatory conditions, the P2Y_2_ receptor has been found to blunt glucose sensitivity, as demonstrated by studies using male P2Y_2_ receptor knockout mice (P2Y_2_R^-/-^) fed a high-fat diet, which showed significantly lower blood glucose at steady state and during a glucose tolerance test (GTT) compared to wild-type mice (4). This implies that the absence or inhibition of the P2Y_2_ receptor activity could enhance glucose tolerance, potentially benefiting individuals with impaired glucose metabolism or insulin resistance.

Glucose regulation is a critical to maintaining energy balance and overall metabolic health. The clearance of glucose from the blood is mainly accomplished through the uptake of glucose by various tissues, particularly skeletal muscle and adipocytes. Insulin, a hormone produced by the pancreas, plays a key role in glucose homeostasis by signaling the translocation of glucose transporter 4 (GLUT4) to the cell surface, promoting glucose uptake by target tissues. Skeletal muscle, being the major site of insulin-mediated glucose disposal, contributes significantly to the regulation of blood glucose levels (5). While insulin dependent trafficking of GLUT4 through the insulin receptor (INSR) is an important regulator of glucose absorption, a dysregulation of GLUT4 and INSR expression has been noted in adipose tissue from P2Y_2_R null mice fed a high fat diet (4). A role for P2Y_2_R receptor activation in regulation of insulin signaling was recently confirmed when activation of P2Y_2_R in hepatocytes disrupted insulin dependent signaling, likely through competition for phosphatidylinositol 4,5-bisphosphate (PIP_2_), an intermediate required for both signaling pathways (6).

Several studies have reported variations in insulin sensitivity, glucose metabolism, and the prevalence of metabolic disorders between males and females (7). Women have a higher insulin sensitivity (8). In mice, females have a greater glucose sensitivity during glucose tolerance testing (9, 10). Hormonal differences, genetic factors, and body composition are among the things that likely contribute to these sex-related disparities. However, the underlying mechanisms and specific receptors involved in the sexual dimorphism of glucose regulation remain incompletely understood. Moreover, there remains limited information regarding the potential sex differences in the role of the P2Y_2_R in glucose homeostasis.

Sex difference in immune responses have been widely observed. For example, human biological females are more susceptible to autoimmune disease (11) and males have higher mortality with COVID-19 infection (12). Various cytokines have been shown to have sex-dependent expression. Female mice have greater circulating cytokines, such as IL-6, IL-10 and TNFα, in response to lipopolysaccharide (LPS) administration (13, 14). LPS, or endotoxin, induces acute inflammatory challenge by binding to toll like receptor 4 (TLR4), initiating the signaling cascade that activates proinflammatory cytokines, and also leads to hypoglycemia (15). Several studies have shown that LPS administration upregulates P2Y_2_R (16–18). TLR4 is expressed at a higher level on macrophages from male mice (13) and is stimulated to a greater extent on macrophages of male mice in the acute stages of viral infection (19) and or after LPS challenge (13).

Despite the emerging understanding of the P2Y_2_ receptor’s impact on glucose homeostasis, inflammation, and the established sex differences in both, there is a notable lack of information regarding sex differences in P2Y_2_R activity *in vivo*. Understanding whether there are sex-specific differences in the P2Y_2_R influence on glucose homeostasis could have important implications for basic research design, but also for designing treatment strategies for metabolic disorders or inflammatory diseases. Here, we provide the first sex-inclusive comparison of glucose tolerance in P2Y_2_R null and wild-type mice, in the unstimulated and acute inflammatory state. We find that the receptor plays a male-specific role in influencing glucose homeostasis in the fasted state and during acute inflammation.

## 2 Materials and methods

### 2.1 Mice

All housing and procedures were approved by institutional animal care and use committee (IACUC). P2Y_2_R heterozygote knockout mice (20) were obtained from Jackson laboratories and backcrossed to C57BL6. The colony was maintained by heterozygous mating. Genotype was confirmed by in a single PCR reaction using the following three primers: AGCCACCCGGCGGGCATAAC (antisense to both wild-type and P2Y_2_R^-/-^), GAGGGGGACGAACTGGGATAC (sense to wild-type only), and AAATGCCTGCTCTTTACTGAAGG (sense to P2Y_2_R^-/-^ only). All experiments were performed on age-matched (8-12 weeks old), co-housed littermates, with pairs of LPS and saline injected mice performed in parallel.

### 2.2 Glucose tolerance testing

Mice were intraperitonially injected with 2 mg/kg LPS or 0.9% saline 24 hours before GTT. Mice were fasted for 5 hours before GTT or sacrifice (for tissue collection). Ten to fifteen minutes before the GTT, lidocaine (lidocaine HCl 2%, Patterson Veterinary Supply, Inc., Kansas City, Missouri) was applied to the tail for local anesthesia. Fasting blood glucose (t = 0 min) was measured immediately before intraperitoneal injections with 2 g/kg dextrose (Patterson Veterinary Supply, #78008986, Kansas City, Missouri). Blood glucose levels were measured from blood samples collected from snipped tails using blood glucose test strips coupled with a glucometer (ReliOn) at 10, 20, 30, 45, 60, 75, and 90 minutes after the dextrose injection.

### 2.3 qRT-PCR

Tissue was extracted from fasting mice that were intraperitonially injected with 2 mg/kg LPS or 0.9% saline 24 hours prior. Total RNA was extracted with Trizol (Invitrogen) and converted to cDNA using high capacity cDNA reverse transcription kit (Applied Biosystems). qPCR was performed using iTaq Universal SYBR green mix (Bio-Rad) and primers for *GLUT4* (tcttattgcagcgcctgag and gagaatacagctaggaccagtg), *P2RY2* (CTTTTTGCTGTGCCCTTTTC and CTGGCCATAAGCTAACA), *INSR* (TCAATGAGTCAGCCAGTCTTC and CAATTCCATCACTACCAGCGT) and *PPIA* (control, caaacacaaacggttcccag and ttcaccttcccaaagaccac). Each reaction was performed in duplicate alongside a no reverse transcriptase control for each sample, and a batch no template control.

Gene expression was analyzed using the Pfaffl method (21). PCR products from each primer set were cloned using p-GEM T-easy (Promega). The average cycle threshold (C_T_) was determined from duplicate qPCR of a dilution series (from 1/10 to 1/1,000,000) of each cloned product. A scatter plot and the trendline was made from the C_T_ average and the log of the dilution. The trendline was R^2^ > 0.99 for all products. The slope of the final trendline was used to determine the efficiency using the equation E = -1 + 10^^(-1/slope)^. To determine the relative gene expression, the Ep value (Ep = E + 1) was used to determine the fold change = (Ep)^ΔCT^, where the ΔC_T_ was calculated by taking the average of each target C_T_ and subtracting that from control average C_T_ from the same target. The fold change for *GLUT4, P2RY2*, and *INSR* was then divided by the fold change for the control, *Ppia*, from the same sample.

### 2.4 Statistics

JASP, the open-source program supported by the University of Amsterdam, was used for all statistical analyses. A classical two-way *ANOVA* was used to analyze the statistics for gene expression, AUC and fasting blood glucose. An outlier test was used to determine outliers, which were removed prior to analysis. Repeated measures ANOVA was used to analyze GTT. Significant main effects (p < 0.05) or large effect sizes (partial eta squared, η^2^_p_) were further explored by Tukey-Mann multiple comparisons or independent t-tests. Statistical methods were reviewed by biostatisticians (R-stats, Missouri State University).

### 2.5 ELISA

After GTT, mice were anesthetized with isoflurane and cardiac punctures are performed to extract the blood. Whole blood was immediately centrifuged at 10,000 rpm at 4 ˚C for 10 minutes to allow separation of plasma and erythrocytes. Plasma was stored at -80 ˚C. IL-6 from plasma was later quantified using the mouse IL-6 Uncoated ELISA kit (Invitrogen), per instructions. All samples were run in duplicates.

### 2.6 Human gene expression

The Genotype-Tissue Expression (GTEx) Project was supported by the Common Fund of the Office of the Director of the National Institutes of Health, and by NCI, NHGRI, NHLBI, NIDA, NIMH, and NINDS. The data used for the analyses described in this manuscript were obtained from the GTEx Portal on 6/7/2023.

## 3 Results

### 3.1 P2Y_2_R regulation of glucose metabolism

**Figure 1:**
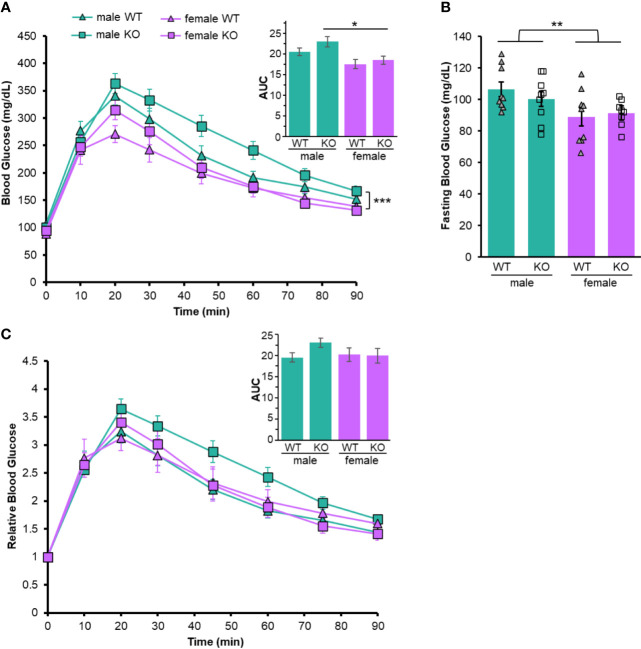
Sex-dependent glucose tolerance in wild-type and P2Y2R^-/-^ mice. **(A)** Glucose tolerance testing. Blood glucose levels after IP injection of glucose in male (green) and female (purple), wild-type (WT, triangles) and P2Y_2_R^-/-^ mice [Knockout (KO), squares]. Repeated measured ANOVA, ***p < 0.001 sex main effect. Inset: Area under the curve from GTT experiments. One way ANOVA, ***p < 0.001 sex main effect, Tukey multiple comparisons, *p < 0.05 (male KO compared to female KO). **(B)** Mean fasting blood glucose from same animals as in **(A)** One way ANOVA, **p < 0.01 effect for sex. **(C)** Each blood glucose level over the course of GTT was normalized to the baseline (time = 0). Repeated measured ANOVA, no significant differences. Inset: Area under the curve from normalized experiments. One way ANOVA, no significant differences. Error bars = SEM. N = 9.

We sought to determine the role of the P2Y_2_ receptor in regulating glucose absorption in mice. Therefore, we initially subjected both wild-type and P2Y_2_R knockout animals (P2Y_2_R^-/-^) to glucose tolerance testing. Blood glucose was lower over the course of GTT in females compared to males in wild-type and P2Y_2_R^-/-^ mice (**Figure 1A**). This is consistent with previous reports indicating that blood glucose levels are lower in female wild type mice, indicating greater insulin sensitivity and glucose clearance relative to males (10). Fasting blood glucose was significantly reduced in females compared to males, regardless of genotype, providing further evidence that fasting glucose levels are sex-dependent (**Figure 1B**). There were no significant differences between wild-type and P2Y_2_R^-/-^ animals of the same sex in GTT or fasting glucose levels (**Figures 1A, B**). However, repeated measures ANOVA showed that blood glucose during GTT in male P2Y_2_R^-/-^ mice was significantly higher than either female group. The area under the curve (AUC) for the GTT further indicates that the males have decreased glucose tolerance compared to either genotype of female mice (**Figure 1A** inset).

Different fasting glucose levels have the potential to skew analysis of glucose tolerance differences between males and females. Therefore, we normalized the blood glucose throughout each individual GTT to the fasting level. After normalizing, GTT glucose levels and AUC showed no significant differences (**Figure 1C**). Therefore, the sex-dependent differences in glucose throughout GTT are influenced mainly by differences in the fasting state. P2Y_2_R plays no discernable role in regulating glucose tolerance at steady state.

### 3.2 P2Y_2_R-dependent glucose regulation during LPS-induced acute inflammation

**Figure 2:**
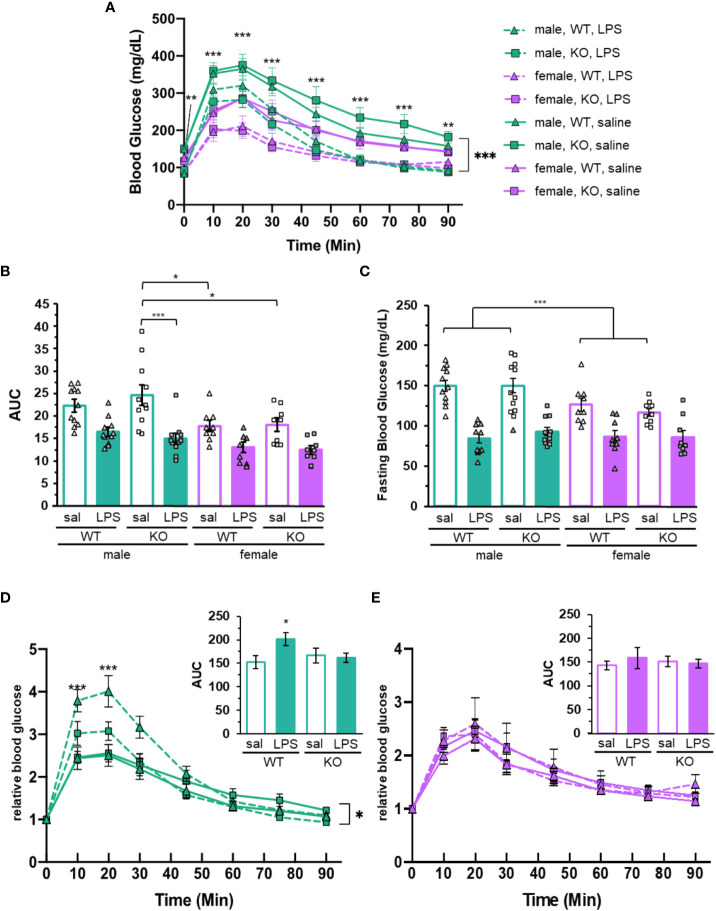
Glucose tolerance and blood glucose is dependent upon sex, P2Y_2_R and LPS treatment. **(A)** Glucose tolerance testing (GTT) of mice injected with LPS (dashed lines) or saline (solid lines) 24 hours before glucose injection (time 0). Blood glucose was determined over 90 min time course. Green lines represent the male mice (N = 11). Purple lines represent female mice (N = 9). Both P2Y_2_R knockout animals (square) and wild-type (triangle) were included. Two-way ANOVA with repeated measures, ***p < 0.001, **p < 0.01 for sex and treatment effects, as well as significant effects at each individual time point. **(B)** Area under the curve (AUC) and **(C)** relative fasting glucose levels from male (green) and female (purple) mice treated with saline (empty) or LPS (filled). Individual data points are represented with squares (P2Y_2_R^-/-^) or triangles (wild-type). Error bars = SEM. **(B)** Two-way ANOVA, Tukey multiple comparisons, ***p < 0.001 and *p < 0.05 for significant difference between indicated groups. **(C)** Two-way ANOVA ***p < 0.001 main effects for treatment and sex. ***p < 0.001 male saline versus female saline. **(D)** Male and **(E)** female GTT data from **(A)** normalize to each individual baseline (fasting, time = 0). Two-way ANOVA with repeated measures, *p < 0.05 (males) treatment effect. ***p < 0.001 treatment effect in wild-type males at indicated time points. Insets: Area under the curve from normalized experiments. One way ANOVA. *p < 0.05 LPS treat males. Error bars = SEM.

To assess the role of the P2Y_2_R in glucose regulation during inflammation, wild-type and P2Y_2_R^-/-^ mice were injected with a moderate dose of LPS to induce an acute inflammatory response. Glucose tolerance tests were conducted 24 hrs after LPS injection. Both male and female mice were studied to provide an inclusive look at the role of P2Y_2_R. Blood glucose levels and AUC indicate that both sex and treatment have significant effects on glucose tolerance (**Figures 2A, B**, respectively). Consistent with previous studies using a similar dose of LPS (18, 22), the treatment induced acute systemic inflammation which included a significant increase in plasma IL-6 (**Supplemental Figure 1**). Also consistent with findings from Nguyen et al. (22), LPS treatment resulted in significant hypoglycemia throughout GTT (p < 0.001; **Figure 2A**). LPS injected mice were hypoglycemic at even the fasting, time 0, suggesting that LPS induced a significant effect on steady-state glucose levels (treatment main effect, p < 0.001; **Figures 2A, C**). Similar to our previous results, fasting glucose levels were sex-dependent in the control mice (saline treated; **Figure 2C**). Specifically, female saline treated mice had a reduced fasting glucose compared to male controls (p < 0.001; **Figure 2D**). There were no sex differences in the fasting glucose with LPS treatment (p = 0.991; **Figure 2D**). Thus, fasting glucose levels are sex-dependent at steady state, but sex differences are eliminated in the presence of LPS-induced acute inflammation.

The area under the GTT curve is reduced in LPS treated animals compared to those treated with saline (treatment main effect p < 0.001; **Figure 2B**), confirming that LPS-induced inflammation improves glucose tolerance in both sexes. While the average AUC for saline treatment is higher than LPS in both males and females, only the P2Y_2_R^-/-^ males were significantly affected by treatment (p = 0.0678 for wild-type males and p < 0.001 for P2Y_2_R^-/-^ males). The AUC for saline treated P2Y_2_R^-/-^ males was increased compared to all females animals, including the P2Y_2_R^-/-^ and wild-type saline treated females (p = 0.0392 and p = 0.024, respectively, **Figure 2B**). This suggests that the P2Y_2_ receptor significantly dampens the LPS-dependent increase in glucose tolerance, specifically in males.

While blood glucose levels throughout GTT were clearly different between sexes and treatments, we noticed that the curves were near parallel (**Figure 1A**), indicating that the differences in baseline blood glucose potentially exacerbate or negate potential differences in the rates of glucose flux. The rate of plasma glucose change is arguably a more representative measure of response to glucose challenge than raw blood glucose. Therefore, we normalized the blood glucose throughout each GTT to its own baseline (fasting blood glucose). Wild-type male mice treated with LPS have significantly increased relative blood glucose compared to any other group at the 10- and 20-min time points, indicating that these mice have a higher amplitude of glucose flux compared to all other groups, including P2Y_2_R^-/-^ male mice (**Figure 2D**). Accordingly, the AUC for male wild-type mice was increased in LPS treated mice compared to saline, while male P2Y_2_R^-/-^ and all female mice had similar AUC with and without treatment (insets, **Figures 2D, E**). There were no differences between genotypes or treatments for female mice after normalizing the blood glucose levels, indicating that the glucose tolerance of females is not affected by LPS or P2Y_2_R (**Figure 2E**). This suggests that P2Y_2_R helps male mice regulate blood glucose in response to challenge.

### 3.3 Gene expression of factors that regulate glucose homeostasis.

**Figure 3:**
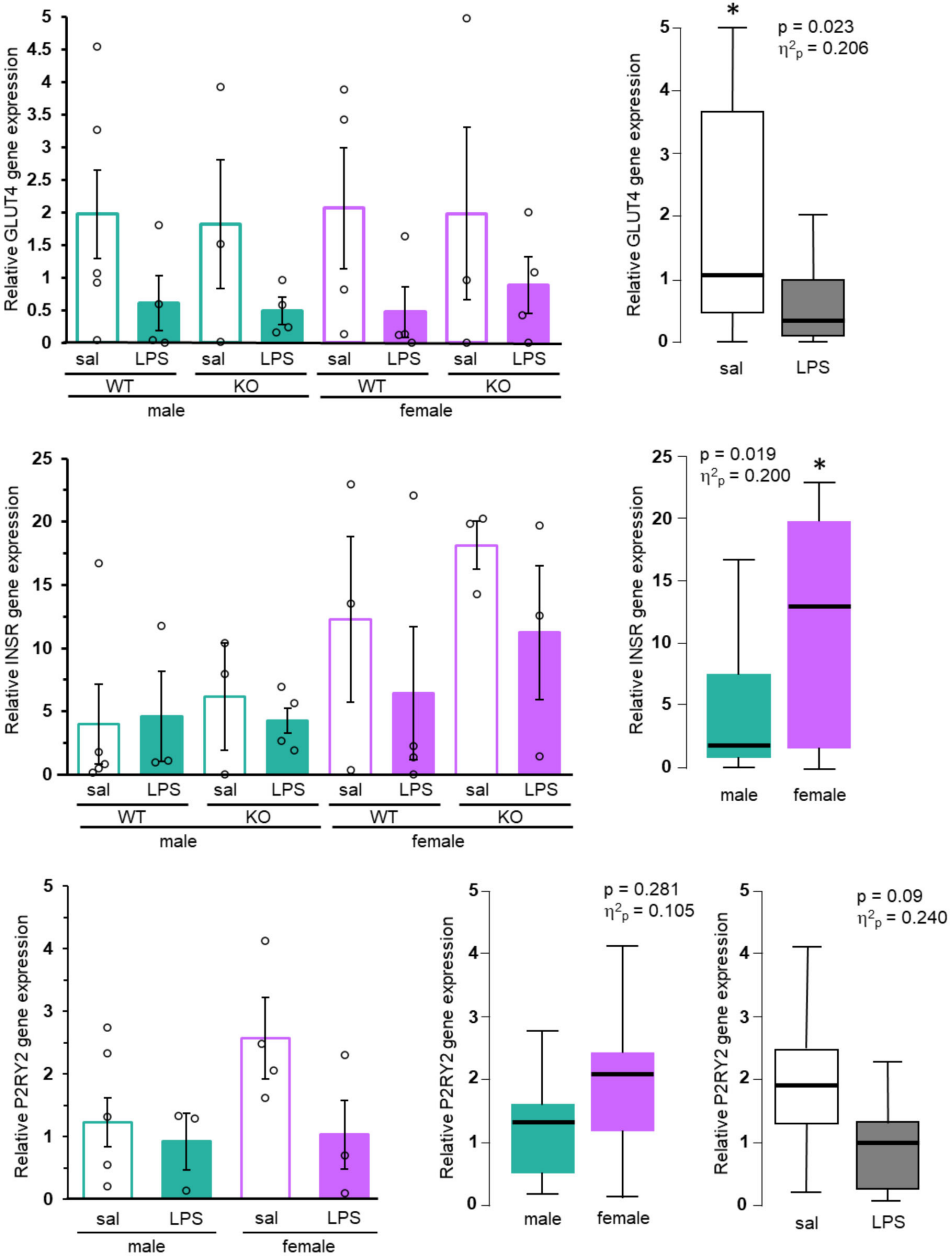
GLUT4, INSR, and P2RY2 gene expression in mice. **(A)** *GLUT4*, **(B)** *INSR*, and **(C)** *P2RY2* levels from muscle of control (saline, empty bars) or treated (LPS, filled bars), male (green) and female (purple) mice were analyzed by qRT-PCR. Mean gene expression (+/- SEM) from each individual group (N ≥ 3) shown on the left. Box and whisker plots of noteworthy main effects are shown on the right. Two-way ANOVA, *p < 0.05. Exact p values and effect sizes are inset into each plot.

When examining expression of both P2Y_2_R and *GLUT4*, a key molecule in glucose uptake, across human tissues using the publicly available GTEx database, both had the highest level of expression in skeletal muscle. Expression in adipose, pancreas and liver, key tissues involved in glucose regulation, was significantly lower than skeletal muscle, indicating that the primary site of their action may be in skeletal muscle (**Supplementary Figures 2A, B**). *GLUT4* gene expression in muscle isolated from fasting mice was determined by qRT-PCR (**Figure 3A**). Statistical analysis identified a significant effect of LPS treatment on *GLUT4* (p = 0.023, **Figure 3**). Several of the samples showed very low or no *GLUT4* mRNA. There were no other significant differences in the expression of *GLUT4*, however, the wide variability of the data and limited sample size suggest that any further conclusions are not possible.

One reason for sex-specific effects of P2Y_2_R on glucose homeostasis may be due to differential expression of the receptor. Expression of the *P2RY2* gene in human skeletal muscle was similar between males and females (TPM = 27.5 and 26.2, respectively; **Supplementary Figure 2B**). Analysis of P2Y_2_R gene expression in muscle of wild-type mice also revealed no significant differences in expression between the sexes (p = 0.281, **Figure 3C**). Previous studies showed that mice fed a high fat diet had increased gene expression of *P2RY2* (4). However, mean *P2RY2* gene expression was lower in LPS treated animals compared to saline. This effect failed to show a significant p value (p = 0.09; **Figure 3C**), however, the calculated effect sizes for LPS treatment (η^2^_p_ = 0.240) indicates a moderate effect, reflecting potentially important decreases in P2Y_2_R expression during LPS treatment.

We also analyzed expression of *INSR* in muscle of fasting mice. Insulin receptor, a key regulator of insulin dependent glucose absorption, was previously shown to be upregulated in P2Y_2_R^-/-^ mice maintained on a high fat diet (4). Females have significantly higher expression of *INSR* than males (p = 0.019, **Figure 3B**). While the mean gene expression in our animals was greater in saline compared to LPS treated animals of both sexes and genotypes, no conclusions can be made regarding treatment due to the limited power and high variability of the data. Human *INSR* expression is much higher in female-specific organs including the ovary, uterus, and fallopian tubes, than in skeletal muscle (**Supplementary Figure 2C**). Gene expression in the muscle of human is similar between sexes [TPM of 19.59 (females) compared to 16.59 (males), **Supplementary Figure 2C**]. Altogether, this suggests that any sex-specific effects of the INSR are primarily due to regional expression rather than muscle-specific expression.

## 4 Discussion

P2Y_2_R plays a male-specific role in regulating glucose metabolism. Male P2Y_2_R deficient animals have reduced tolerance to glucose administration compared to wild-type males, and all female mice (**Figures 1A, C**), suggesting that the receptor plays an important role in glucose sensitivity in males. The rates of glucose excursion, as well as any difference in tolerance are negated when GTT data is normalized to fasting (**Figure 1C**), indicating that the role P2Y_2_R plays in regulation of glucose is primarily at the fasting level. Female mice had lower glucose at fasting and throughout GTT compared to male mice (**Figure 1**). This is consistent with some past work (9, 10, 23) but not all (24). We show that LPS administration improved glucose tolerance in all mice, across sex and genotype (**Figure 2**). Inflammation induces a decreased glucose excursion time in different inflammatory models, whether glucose was administered via intraperitoneal injection (25) or via oral gavage (26). Male mice may have increased levels of the LPS receptor TLR4 during the acute stages of inflammation (13, 19), and the cytokine response to LPS administration is sexually dimorphic (13, 14). However, these factors did not result in significant sex-dependent glucose response (**Figure 2E**) in our LPS-treated animals. One way to explain the reduced blood glucose in females is through the upregulation of GLUT4. We did not detect significant sex-dependent levels of *GLUT4* mRNA, however, we did detect significantly more *INSR* expression in females (**Figures 3A, B**). Insulin signaling through the insulin receptor can trigger the translocation of GLUT4 protein on internal vesicles to the plasma membrane, promoting glucose uptake independent of transcription levels. Thus, it is possible that sex-dependent fasting blood glucose levels are at least partially dependent upon insulin signaling within the muscle. Importantly, our data does not rule out differential gene expression or insulin signaling in other tissues or cell types. Our gene expression analysis is only preliminary and more investigation is needed to determine if local sex-dependent expression of *P2Y_2_R, GLUT4* and/or *INSR* could be responsible for the observed changes in glucose homeostasis.

Sex differences in fasting glucose (**Figures 1B**, **2B**) were eliminated in the presence of LPS, suggesting that inflammation overrides P2Y_2_R-dependent control of glucose sensitivity. The LPS-dependent reduction in fasting blood glucose (**Figure 2**) could be due to increased *GLUT4* translocation to the surface during inflammation in skeletal muscle, but could also be influenced by increased glucose absorption in the liver or adipose tissue. The majority (80-90%) of glucose is absorbed by skeletal muscle (5), but alterations in glucose regulation in the liver or adipose tissue cannot be ruled out.

The P2Y_2_ receptor was previously found to play a role in high fat diet-related obesity, a condition marked by activation of inflammatory pathways. P2Y_2_R knock out animals have increased glucose tolerance (4) and improved insulin sensitivity (4, 27), compared to wild-type mice fed a high fat diet. The loss of the receptor globally (4) and myeloid-specific loss decreases the inflammatory response (18). Consistent with previous research, here we show that the P2Y_2_R^-/-^ is protective against inflammation-stimulated effects. The sex-dependence and inflammation-dependence of blood glucose largely masked the effect of the P2Y_2_ receptor on glucose excursion time. However, after normalizing glucose levels to baseline, it was apparent that the P2Y_2_R deficient male mice are protected from inflammation-mediated effects on glucose tolerance. While the wild-type males had a reduced tolerance to glucose, the P2Y_2_R^-/-^ males were similar to the females, suggesting that the P2Y_2_R has a specific effect in male mice that increases their tolerance. Interestingly, a previous study testing only female mice noticed that those lacking P2Y_2_R on myeloid cells had similar glucose levels and tolerance to wild-type mice in the presence of a HFD-induced obesity (18). This is in contrast to male whole body P2Y_2_R^-/-^ mice on HFD that showed decreased blood glucose and increased glucose tolerance (4). While the models in the two studies are different, they support our work suggesting that P2Y_2_R role in glucose homeostasis is more prominent in males. The resistance of females to alteration in glucose tolerance and reduced effects of LPS on baseline glucose levels may be due, in part, to greater insulin-sensitivity in females (28). Our own data suggests that there are greater levels of *INSR* in female skeletal muscle, compared to that of males, supporting the idea that females may be more insulin-sensitive.

It is clear that P2Y_2_R plays a more important role in glucose regulation during LPS-stimulated inflammation in fed males than it does females. The underlying differences between the sexes might be due to differential inflammatory signaling. Sex-dependent differences in cytokine production and inflammatory factors have been widely observed. However, females mice are less susceptible to insulin resistance triggered by high fat diet (29), thus this male-specific effect is not isolated to LPS-treatment, but is more widely observed across inflammatory models. We show no significant differences in P2Y_2_R expression between male and female mice (**Figure 3C**) and data from humans also suggests that P2Y_2_R is expressed similarly between males and females (**Supplementary Figure 2**), thus, differential expression of P2Y_2_R cannot explain the dimorphic dependence on the receptor. The P2Y_2_R gene expression profile in humans is similar to *GLUT4* expression in that there is a high level of expression in the skeletal muscle, but low levels of expression in adipose, liver and sex-specific tissues such as the testes and ovaries. This suggests that the convergence of P2Y_2_R signaling, and glucose sensitivity may occur in the muscle. Previous studies showed that *P2Y_2_R* expression was increased by LPS treatment in cultured cells (16, 17) and in the blood (18), however, our study did not find significant induction of *P2Y_2_R* in the muscle with LPS-treatment. While effect sizes suggest a possible important effect of sex and LPS treatment on *P2Y_2_R* gene expression, more data is be needed to rule out LPS-dependent expression of *P2Y_2_R* in muscle of mice. It is possible that *P2Y_2_R* induction is cell-type specific, and that P2Y_2_R signaling can exert its effects over long ranges. It is also possible that the dose of LPS used here was not sufficient to induce *P2Y_2_R* expression in the muscle. However, plasma IL-6 (**Supplementary Figure 1**) and expected LPS-dependent effects on fasting glucose levels (**Figure 2**) confirm that the dose and timing of LPS used here was sufficient to stimulate systemic inflammation.

While sex-dependent regulation of glucose metabolism seems to be isolated to steady-state, the role of the P2Y_2_ receptor is most prominent during glucose challenge, affecting both the amplitude and rate of glucose accumulation. P2Y_2_ receptor signaling may interact with the insulin signaling pathway to affect the ability of insulin to signal glucose uptake in peripheral tissues, making the P2Y_2_R^-/-^ males less insulin sensitive. A recent study showed that, in cultured hepatocytes, P2Y_2_ activation reduced insulin-dependent signaling, ultimately reducing glucose uptake there (6), illustrating that P2Y_2_R and insulin signaling pathways intersect to regulate glucose homeostasis locally. Of course, P2Y_2_R may also reduce the levels of insulin released during glucose challenge. The role of receptor may be isolated to males due to expression of metabolism gene(s) from sex chromosomes, or perhaps by androgen-regulation or estrogen-repression of P2Y_2_R activity. Indeed, sex differences in glucose regulation in HFD mouse models is minimized by gonadectomy, though androgens were primarily responsible for sex-differences (30). Testosterone also shown to decrease the levels of IL-6 in response to LPS administration in mice, providing more suggestion that the male sex hormones may be the key to understanding sexual dimorphism in glucose regulation (31).

The purinergic receptor plays important roles in inflammation and in glucose regulation, though its role appears to be more critical in males, at least among animals in the young adult age rage (8-12 weeks) tested here. Overall, this work highlights the sexual dimorphic nature of glucose regulation, but also displays a new sex-specific role for purinergic signaling. Studies that systematically address sex-dependent regulation provide crucial insights into an inclusive understanding of physiological processes, dysregulation, and treatment. Indeed, recent evidence suggests that metformin, a common pharmaceutical treatment for insulin resistance, may exert its regulation primarily through P2Y_2_R (6). P2Y_2_ receptor agonist have been approved to treat dry eye in some countries, and purinergic signaling is an enticing drug target for treatment of many conditions, including immune and infection-related disease (32). As these therapeutic treatments become reality, sex may be an important consideration in clinical trials and in clinical practice.

## Data availability statement

The raw data supporting the conclusions of this article will be made available by the authors, without undue reservation.

## Ethics statement

The animal study was approved by Missouri State Institutional Animal Care and Use Committee. The study was conducted in accordance with the local legislation and institutional requirements.

## Author contributions

RU and JW were the main advisors on the project, overseeing experimental design, data analysis and manuscript preparation. RU and SZ drafted the manuscript. CR, HM, DJ, NJ and ES collected GTT data and harvested tissues. CR, RU and HM analyzed GTT data. JM collected INSR gene expression data while RU collected and analyzed all other gene expression data. All authors contributed to the article and approved the submitted version.
