# Supplementary figures and images for: Sex-specific effect of P2Y2 purinergic receptor on glucose metabolism during acute inflammation
Source: Front Endocrinol (Lausanne). 2023 Aug 28;14:1248139. doi: 10.3389/fendo.2023.1248139 (PMC10494456; doi:10.3389/fendo.2023.1248139)

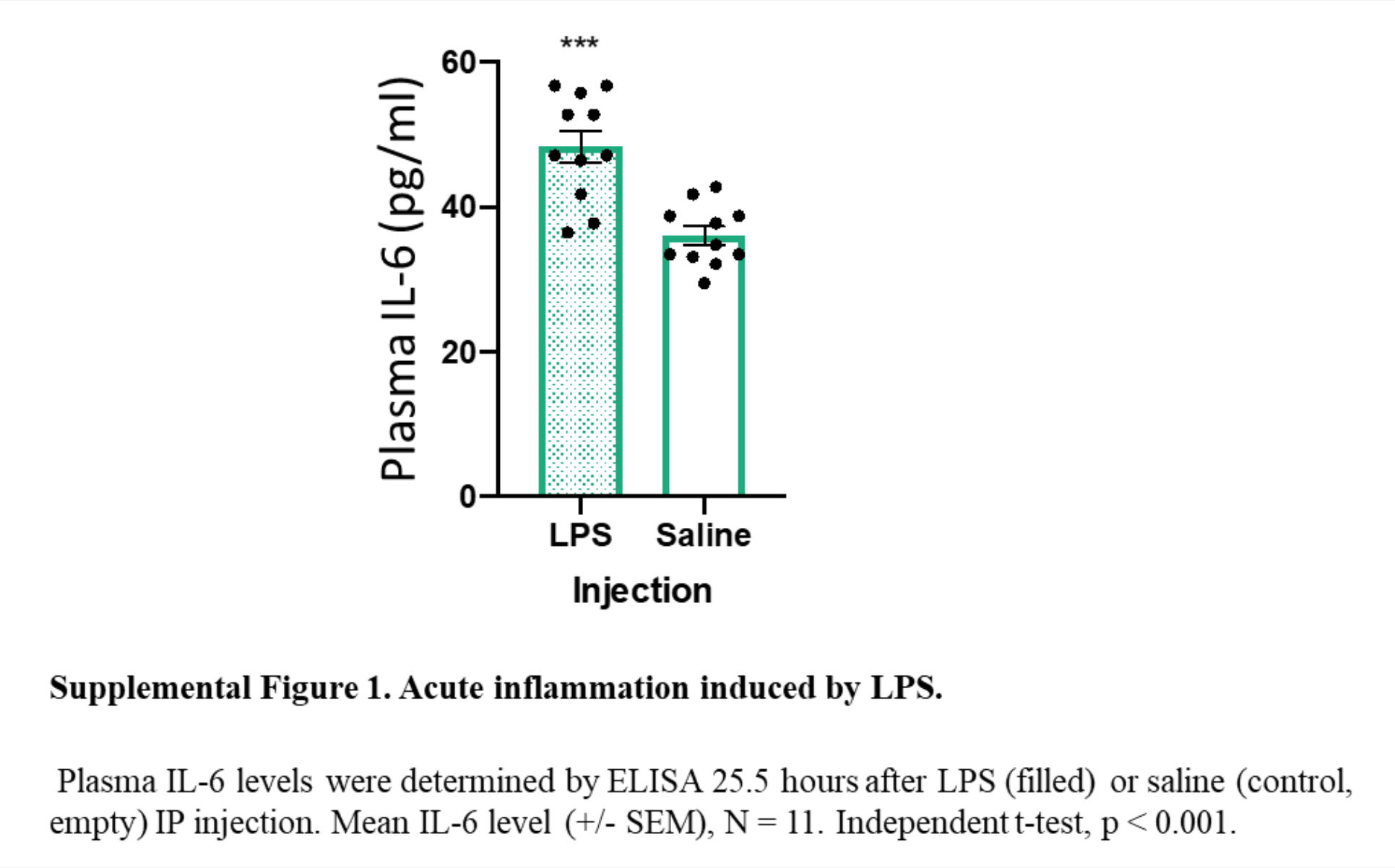

Supplement: Supplementary file 1 [file Image_1.jpeg]

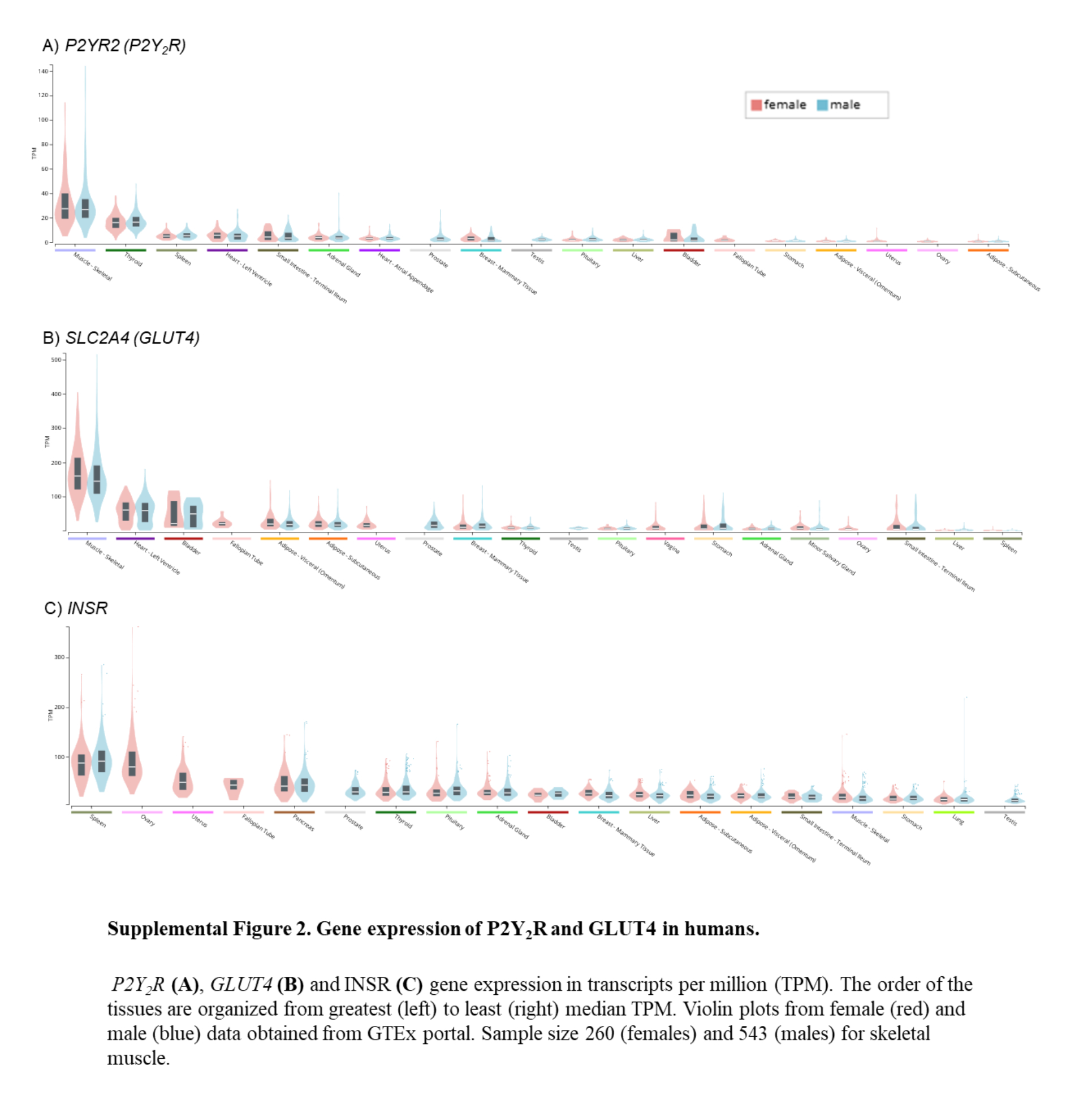

Supplement: Supplementary file 2 [file Image_2.jpeg]
